# A model of proximate protection against pathogenic infection through shared immunity

**DOI:** 10.1128/mbio.03046-24

**Published:** 2024-11-11

**Authors:** Douglas F. Nixon, Margarita Kyza-Karavioti, Sreeradha Mallick, Lillia Daley, Nathaniel Hupert, Nathaniel D. Bachtel, Ioannis Eleftherianos

**Affiliations:** 1 Institute of Translational Research, The Feinstein Institutes for Medical Research, Manhasset, New York, USA; 2 Infection and Innate Immunity Laboratory, Department of Biological Sciences, The George Washington University, Washington, DC, USA; 3 Division of Epidemiology, Department of Population Health Sciences, Weill Cornell Medicine, Cornell University, New York, New York, USA; 4 Department of Medicine, Weill Cornell Medicine, Cornell University, New York, New York, USA; 5 Department of Immunobiology, Yale University School of Medicine, New Haven, Connecticut, USA; University of California, Davis, Davis, California, USA

**Keywords:** *Drosophila*, bacterial infection, immunity, shared protection, shared immunity

## Abstract

*Drosophila melanogaster* exhibits innate immune priming, a mechanism leading to protection upon repeated challenge with a given pathogen. However, whether immunological priming can be propagated from a challenged host to naive bystanders is unknown. Here, we show that priming half a vial of *D. melanogaster* adult flies with non-pathogenic *Escherichia coli* bacteria leads to protection of the whole vial from a lethal dose of the insect pathogen, *Photorhabdus luminescens*. The protective effect observed in these bystander flies was similar in magnitude to that of the *E. coli* primed hosts themselves but did not require transfer of *E. coli* to occur. This work broadens the scope of how immunological priming can occur and suggests that infected hosts can produce signals that influence immunity in their neighbors, leading to a shared immune collective.

**IMPORTANCE:**

Here, we have introduced the new concept of shared immunity and priming by proximity. These findings are of particular significance because they indicate that the presence of compromised hosts can increase the response to the pathogenic challenge of healthy individuals that cohabitate within close distance. This shared immunity may involve proximate boosting of the host’s immune defenses via the sensing of specific chemical, behavioral, or microbial signals. Determining the breadth, mechanistic basis, and translatability of these findings has the potential to transform biomedical research and public health.

## OBSERVATION

Organisms display diverse sensing strategies to detect noxious agents in their environments and initiate appropriate defenses. Immune cells utilize pattern recognition receptors to directly sense conserved molecular features of pathogens, whereas additional modalities detect damage signals released after cellular injury (1). Receptors utilized in olfaction or taste instead recognize molecular cues indicative of spoilage or toxicity and initiate pathogen avoidance prior to downstream post-ingestive signals (2). A unique utilization of such recognition strategies may be found in social insects, such as ants and honeybees, where infected members of the colony are identified, groomed of parasites, or, if too sick, excluded and eliminated (3, 4). These social-ecological studies suggest that the detection of infected hosts creates a shared immunological perception and led us to the hypothesis that the sensing of an infected organism could be transmitted to bystanders providing a new type of shared protection.

**Fig 1:**
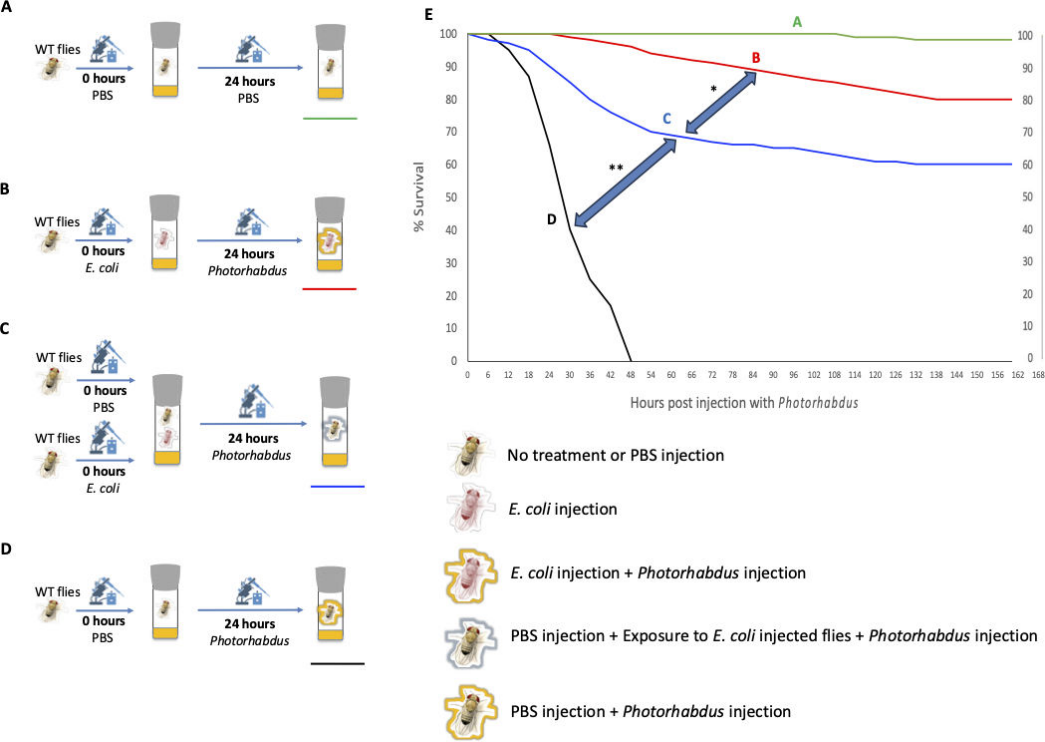
Exposure of naive *Drosophila melanogaster* adult flies to flies infected with a non-pathogenic bacterium provides protection against subsequent infection with a potent bacterial pathogen. (**A**) Injection of *D. melanogaster* wild-type female adult flies with PBS and subsequent injection with PBS. (**B**) Injection of wild-type flies with 10,000 CFUs of the *Escherichia coli* non-pathogenic strain K-12, and 24 hours later injection with 500 CFUs of the insect pathogenic bacterium *Photorhabdus luminescens* strain TTO1. (**C**) Injection of wild-type flies with PBS or 10,000 CFUs of *E. coli* K-12, incubation of the two fly groups in the same vial for 24 hours, and subsequent injection of the PBS-injected group with 500 CFUs of *P. luminescens* TTO1. (**D**) Injection of *D. melanogaster* wild-type flies with PBS, and 24 hours later injection with 500 CFUs of *P. luminescens* TTO1. (**E**) Survival results for the four experimental treatments. The experiment was replicated five times, and in each experiment, 50 adult flies were used per experimental condition (Mantel-Cox, *P < 0.05; **P < 0.01).

*Drosophila melanogaster* is a genetically tractable insect model with well-characterized anti-pathogen immune responses (5, 6). We postulated that the presence of infected adult flies may confer enhanced protection to naive individuals following the challenge with the potent insect pathogen *Photorhabdus luminescens* (7, 8). To test this, we injected Oregon wild-type adult flies with phosphate-buffered saline (PBS) or 10,000 colony-forming units (CFUs) of the non-pathogenic K-12 strain of *Escherichia coli* resuspended in PBS, incubated the two groups of flies in the same vial, and 24 hours later injected both groups of flies with 500 CFUs of *P. luminescens* (Fig. 1; Fig. S1). As expected, dual injection of flies with PBS (Fig. 1A; Fig. S1A) caused no fly mortality (Fig. 1E; Fig. S1E). Also, as expected, injection of a small number of cells of the insect pathogen *P. luminescens* following PBS administration (Fig. 1D; Fig. S1D) caused 100% mortality within 48 hours (Fig. 1E; Fig. S1E). In contrast, injection of PBS instead of *P. luminescens* caused no fly mortality (Fig. S2). We further found that priming adult flies with a large number of live *E. coli* cells (Fig. 1B; Fig. S1B) conferred protection against a secondary infection with pathogenic *P. luminescens*, in line with prior reports (9, 10) (Fig. 1E; Fig. S1E). We also found that 60% of the unprimed flies (i.e., that had received only PBS injections without any live *E. coli* cells) that were confined in the same living space as flies inoculated with *E. coli* (Fig. 1C; Fig. S1C) were able to survive a subsequent infection with *P. luminescens* (Fig. 1E; Fig. S1E). One could postulate that this effect could be due to *E. coli* transfer from inoculated flies to those injected with PBS in the same vial. However, PBS-injected flies that were homogenized after being confined for 24 hours with *E. coli*-injected flies did not grow any *E. coli* (Table S1). Therefore, we conclude that exposed unprimed flies were indirectly protected via cohabitation with primed flies, though by what means this protection was mediated (e.g., by biochemical signal [such as volatile or contact-transferred molecule] or physical stimulus [such as acoustic, magnetic, or electrical trigger]) is not known.

Further epidemiological and biological characterization of this protective phenomenon should investigate, at a minimum, whether there are threshold effects (i.e., whether there is a particular ratio of susceptible to infected cohabitants above or below which this effect dissipates); whether a particular transmissible element or physical property (not excluding sound and/or electrical phenomena) can be isolated from the environment in which the effect occurs; and whether the priming effect transcends or is specific for the type of infectious agent initially encountered. This shared immunity appears to be a new defensive modality that may, in the right settings, involve proximate boosting of the host’s immune response. Future work will focus on characterizing whether the observed “indirect protective” effect is associated with reduced pathogen load and changes in immunity in the protected flies. Another priority will be to identify whether the shared protection phenotype instead relates to a modifiable defensive behavior (regulated by, e.g., neuronal control). Elucidating the means of transfer of this protection—from inoculated to unprimed (or “naive”) flies—may help to discriminate between these possibilities.

Our findings are of particular significance because they indicate for the first time that the presence of compromised hosts can boost the response to the pathogenic challenge of healthy individuals that are in close proximity. Together, we have introduced here the new concept of shared immunity and priming by proximity. Determining the breadth, mechanistic basis, and translatability of these observations has the potential to transform both biological science and public health.

## Data Availability

All data are included in the manuscript and the methods in the supplemental material.

## SUPPLEMENTAL MATERIAL

Supplemental Material mbio.03046-24-s0001.docx Supplemental methods, figure, and table.

ASM does not own the copyrights to Supplemental Material that may be linked to, or accessed through, an article. The authors have granted ASM a non-exclusive, world-wide license to publish the Supplemental Material files. Please contact the corresponding author directly for reuse.

## References

[1] Janeway CA, Medzhitov R. 2002. Innate immune recognition. Annu Rev Immunol 20:197–216. doi:10.1146/annurev.immunol.20.083001.084359 11861602

[2] Korn LL, Kutyavin VI, Bachtel ND, Medzhitov R. 2024. Adverse food reactions: physiological and ecological perspectives. Annu Rev Nutr 44:155–178. doi:10.1146/annurev-nutr-061021-022909 38724028 PMC12372117

[3] Casillas-Pérez B, Boďová K, Grasse AV, Tkačik G, Cremer S. 2023. Dynamic pathogen detection and social feedback shape collective hygiene in ants. Nat Commun 14:3232. doi:10.1038/s41467-023-38947-y 37270641 PMC10239465

[4] Baracchi D, Fadda A, Turillazzi S. 2012. Evidence for antiseptic behaviour towards sick adult bees in honey bee colonies. J Insect Physiol 58:1589–1596. doi:10.1016/j.jinsphys.2012.09.014 23068993

[5] Lemaitre B, Hoffmann J. 2007. The host defense of Drosophila melanogaster. Annu Rev Immunol 25:697–743. doi:10.1146/annurev.immunol.25.022106.141615 17201680

[6] Yu S, Luo F, Xu Y, Zhang Y, Jin LH. 2022. Drosophila innate immunity involves multiple signaling pathways and coordinated communication between different tissues. Front Immunol 13:905370. doi:10.3389/fimmu.2022.905370 35911716 PMC9336466

[7] Waterfield NR, Ciche T, Clarke D. 2009. Photorhabdus and a host of hosts. Annu Rev Microbiol 63:557–574. doi:10.1146/annurev.micro.091208.073507 19575559

[8] Eleftherianos I, ffrench-Constant RH, Clarke DJ, Dowling AJ, Reynolds SE. 2010. Dissecting the immune response to the entomopathogen photorhabdus. Trends Microbiol 18:552–560. doi:10.1016/j.tim.2010.09.006 21035345

[9] Pham LN, Dionne MS, Shirasu-Hiza M, Schneider DS. 2007. A specific primed immune response in Drosophila is dependent on phagocytes. PLoS Pathog 3:e26. doi:10.1371/journal.ppat.0030026 17352533 PMC1817657

[10] Prakash A, Fenner F, Shit B, Salminen TS, Monteith KM, Khan I, Vale PF. 2024. IMD-mediated innate immune priming increases Drosophila survival and reduces pathogen transmission. PLoS Pathog 20:e1012308. doi:10.1371/journal.ppat.1012308 38857285 PMC11192365

