# Supplementary material for: A model of proximate protection against pathogenic infection through shared immunity
Source: mBio. 2024 Nov 11;15(12):e03046-24. doi: 10.1128/mbio.03046-24 (PMC11633142; doi:10.1128/mbio.03046-24)
Supplement: Supplemental Material — Supplemental methods, figure, and table. [file mbio.03046-24-s0001.docx]

A model of proximate protection against pathogenic infection through shared immunity

Douglas F. Nixon, Margarita Kyza-Karavioti, Sreeradha Mallick, Lillia Daley, Nathaniel Hupert, Nathaniel D. Bachtel, and Ioannis Eleftherianos

**1. Supplemental Figures**


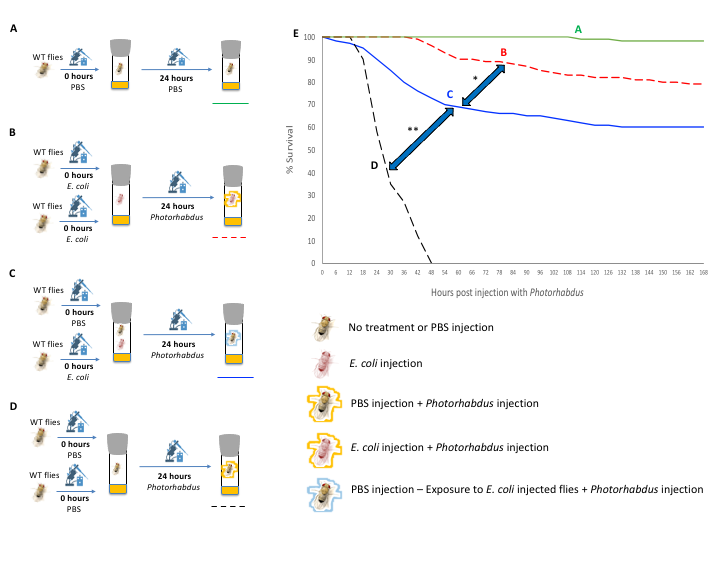


**Figure S1. Exposure of naïve *Drosophila melanogaster* adult flies to flies infected with a non-pathogenic bacterium provides protection against subsequent infection with a potent bacterial pathogen (additional controls).** (A) Injection of *D. melanogaster* wild-type female adult flies with Phosphate-Buffered Saline (PBS) and subsequent injection with PBS, (B) injection of two batches of wild-type flies with 10,000 colony forming units (CFUs) of *Escherichia coli* non-pathogenic strain K-12, incubation of the injected flies in the same vial for 24 hours and injection with 500 CFUs of the insect pathogenic bacterium *Photorhabdus luminescens* strain TTO1, (C) injection of *D. melanogaster* wild-type flies with PBS or 10,000 CFUs of the *E. coli* K-12, incubation of the two fly groups in the same vial for 24 hours, and subsequent injection of the PBS-injected flies with 500 CFUs of *P. luminescens* TTO1, (D) injection of two batches of wild-type flies with PBS, incubation of the injected flies in the same vial for 24 hours and subsequent injection with 500 CFUs of *P. luminescens* TTO1, (E) survival results for the four experimental treatments. The experiment was replicated five times and in each experiment 50 adult flies were used per experimental condition. (Mantel-Cox, * p < 0.05; ** p < 0.01).

**Figure S2. Survival of *Drosophila melanogaster* flies in the absence of *Photorhabdus luminescens* infection (negative controls).** Control treatments involved: A) Injection of *D. melanogaster* wild-type female adult flies with Phosphate-Buffered Saline (PBS) and 24 hours later a secondary injection with PBS, B) injection of wild-type flies with 10,000 CFUs of the *Escherichia coli* non-pathogenic strain K-12 and 24 hours later injection with PBS, (C) injection of wild-type flies with PBS or 10,000 colony forming units (CFUs) of *E. coli* K-12, incubation of the two fly groups in the same vial for 24 hours, and subsequent injection with PBS, (D) injection of two groups of wild-type flies with PBS, incubation of the two fly groups in the same vial for 24 hours, and 24 hours later a secondary injection with PBS, (E) injection of two groups of wild-type flies with *E. coli* K-12, incubation of the two fly groups in the same vial for 24 hours, and 24 hours later injection with PBS. The experiment was replicated five times and in each experiment 50 adult flies were used per experimental condition. All five survival lines are superimposed.

**2. Supplemental Table**

**Table S1.** Bacterial cells are not transferred from *Escherichia coli* injected *D. melanogaster* female adult flies to PBS injected flies when the two groups of flies coexist in the same vial for 24 hours.

|  | Injections | | |
| --- | --- | --- | --- |
| 1^st^ fly group | *E. coli* | PBS | PBS |
| 2^nd^ fly group | N/A | N/A | *E. coli* |
| *E. coli* colonies observed in group 1 flies | + | - | - |

**3. Supplemental Experimental Procedures**

*Fly stocks*

The *D. melanogaster* wild-type Oregon line was used in all experiments. Flies were reared on ready-made fly food (LabExpress, Ann Arbor, MI, United States) supplemented with yeast (Carolina Biological Supply, Burlington, NC, United States). All vials were maintained in an incubator at 25°C and 12 h light/12 h dark photoperiod cycle. The Oregon line was amplified for experimentation by transferring adult flies to fresh vials every third day. Female flies were selected from the same generation and randomly assigned to experimental groups.

*Bacterial stocks*

The bacteria *Photorhabdus luminescens* subsp. *laumondii* (strain TTO1) and *Escherichia coli* (strain K-12) were used in the study. The bacteria were grown in 10 ml sterile LB media for 18 h (*E. coli*) and 22 h (*P. luminescens*) in a shaking incubator set at 30 °C and 210 rpm. Prior to microinjections, the bacteria were washed three times and then diluted in a sterile environment using phosphate-buffered saline (1x PBS, pH 7.4). To inject approximately 500 cells of *P. luminescens* and 10,000 cells of *E. coli*, the optical density (600 nm) was adjusted to 0.100 and 1, respectively, using the Nanodrop 2000c spectrophotometer (Thermo Fisher Scientific, USA).

*Fly microinjections*

*Drosophila melanogaster* Oregon female adult flies of 7–10 days old were used in the experiments. Injections were performed using glass capillaries that were made using the micropipette puller Model P-1000 (Sutter Instruments). The capillaries were used with a microinjector (Nanoject III -Drummond Scientific Co., USA) to inject the flies with sterile 1xPBS buffer (septic injury control), *E. coli* or *P. luminescens*. Flies were anesthetized with carbon dioxide and injected intrathoracically with 18.4 nL of buffer only (control) or bacterial cells resuspended in buffer. Flies that were first injected with PBS and then they were exposed to flies that had previously been injected with *E. coli* were marked with a blue color nail polish. All injected flies were maintained at 25 °C and their survival was monitored every 6 h and up to 1-week post-injection. The fly survival experiment

was replicated five times with different batches of flies and freshly prepared bacteria.

*Data analysis*

Fly survival assay results represent the means from five independent experiments and differences between survival curves (percentage of fly death) were estimated using the log-rank Mantel-Cox. P values below 0.05 were considered significant. All statistical analyses were performed using GraphPad Prism7 software.
